# Microbial Biotreatment of Actual Textile Wastewater in a Continuous Sequential Rice Husk Biofilter and the Microbial Community Involved

**DOI:** 10.1371/journal.pone.0170562

**Published:** 2017-01-23

**Authors:** Jörgen Forss, Markus V. Lindh, Jarone Pinhassi, Ulrika Welander

**Affiliations:** 1 Faculty of Technology, Linnæus University, Växjö, Sweden; 2 Centre for Ecology and Evolution in Microbial model Systems, Linnæus University, Kalmar, Sweden; Fujian Agriculture and Forestry University, CHINA

## Abstract

Textile dying processes often pollute wastewater with recalcitrant azo and anthraquinone dyes. Yet, there is little development of effective and affordable degradation systems for textile wastewater applicable in countries where water technologies remain poor. We determined biodegradation of actual textile wastewater in biofilters containing rice husks by spectrophotometry and liquid chromatography mass spectrometry. The indigenous microflora from the rice husks consistently performed >90% decolorization at a hydraulic retention time of 67 h. Analysis of microbial community composition of bacterial 16S rRNA genes and fungal internal transcribed spacer (ITS) gene fragments in the biofilters revealed a bacterial consortium known to carry azoreductase genes, such as *Dysgonomonas*, and *Pseudomonas* and the presence of fungal phylotypes such as *Gibberella* and *Fusarium*. Our findings emphasize that rice husk biofilters support a microbial community of both bacteria and fungi with key features for biodegradation of actual textile wastewater. These results suggest that microbial processes can substantially contribute to efficient and reliable degradation of actual textile wastewater. Thus, development of biodegradation systems holds promise for application of affordable wastewater treatment in polluted environments.

## Introduction

Water pollution from textile production is intense in nations with large textile industries, such as India, China and Bangladesh. To minimize the negative impact on wastewater from such water demanding industries, it is critically important to decrease pollution, especially from textile dyes like azo and anthraquinone typically recalcitrant to degradation. The level of pollution including azo and anthraquinone dyes can be vast; for example, Indian authorities closed the textile industry in Tirapur in 2011 as a result from excessive water pollution [1]. Moreover, it is notable that a significant portion of the textile industries is located in developing countries, where wastewater treatment is often an expensive option and requires detailed knowledge. Therefore it is necessary to integrate cost-efficient and simple treatment systems into textile production. Efforts to develop such novel, affordable and applicable degradation systems have been aided by investigating biodegradation performed by naturally occurring microbial communities in biofilters [2–7]. Color can be removed from the process water by microbial adsorption or degradation [5]. Nevertheless, degradation is preferable as the color is removed, and the microbial process is sustainable over time. In contrast, adsorption would saturate a continuous process after some time, while microbial degradation would adapt and increase the effectiveness of microbial decolorization over time.

Currently, there are approximately thirty different bacterial families isolated from different dye degradation experiments or investigations involving dye degradation [8]. Interestingly, several of the bacteria identified in these studies have genes for different azoreductases [5]. For example, Khalid, Kausar [9] isolated several bacteria from seawater sediments that showed potential to degrade reactive dyes at high salinity. In fact, both Dafale, Wate [10] and Saratale, Saratale [5] emphasize that biodegradation with natural microbial consortia is a promising approach, capable of degrading a broader range of dyes and metabolites such as aromatic amines compared to a single cultivated species. In addition, biofilm processes degrade metabolites to a higher extent [11], has better resistance to fluctuations in loads, starvation periods and washouts than active sludge treatment [12].

Although fungi are considered to be important decomposers of e.g. large complex organic molecules, and can interact with bacteria in degrading organic matter, the presence and role of naturally occurring fungi in wastewater is largely unknown [13]. Still, Yang, Wang [6] found several fungi in a full scale dyeing wastewater treatment. More recently Punzi, Anbalagan [3] identified fungi in their treatment of Remazol Red in anaerobic bioreactors. Moreover, different white-rot fungi have been widely investigated in the field of dye degradation due to their extracellular lignolytic enzymes, e.g. peroxidase, ligninase and laccase [14, 15]. Collectively, these studies highlight the presence of specific bacteria and fungi adapted to wastewater environments carrying the physiological potential to degrade large organic molecules. Thus, efforts to investigate microbial composition and processes in experimental biofilters are highly valuable for developing simple, affordable and applicable biodegradation systems.

Nevertheless, most studies on dye-degrading microbes have been performed under ideal culture conditions, while the influence of complex mixtures of process chemicals on degradation and microbial community composition is poorly understood [8]. In addition to dyes, textile wastewater is also characterized by a complex matrix of chemicals, with varying pH, Biology Oxygen Demand (BOD) and Chemical Oxygen Demand (COD) depending on materials and processes. For example, Bisschops and Spanjers [16] report BOD values between 380–2020 mg L^-1^ and COD values ranging from 2600 to 11500 mg L^-1^ for cotton processes. Much of the BOD and COD originate from fats, waxes and fibers used and washed off during the process. Moreover, salts are used to enhance the adherence of dyes to the textile fiber. pH can vary from 5.5 to 11.6 in different systems [17] and the salinity can range from 250–63750 μS/cm in textile process waters [18]. These environmental factors put particular demands on the microorganisms living in textile wastewater environments. In particular, bacteria are affected by both variations in salinity and pH, with optimal salinity between 0.05–1.6% (100–26000 μS/cm) [19, 20] and pH conditions typically between 7–9.5 [5, 21].

To determine the adaptability of the microbial community in biodegradation of textile dyes and evaluate the prospect of implementation, it is essential to investigate microbial performance in actual wastewater that includes all process chemicals [12]. Even though there are methods to treat textile wastewaters, textile wastewaters are still released in many rivers in developing countries due to lack of capital to invest in treatments [18, 22, 23]. This is especially relevant for textile mills producing fabrics for the local markets. In the present paper a continuous sequential biofilter system was constructed, where indigenous microorganisms provided by and supported by rice husks were used for biodegradation of actual textile wastewater. Rice husks have the advantage of a high content of silica, are resistant against degradation [24] and are available in most developing countries. Furthermore, it releases few compounds to the water and can be preserved for a long time. To evaluate if a microbial community capable of degrading the substances develops in the highly competitive environment a wastewater treatment facility constitutes. Real wastewater biodegradation studies are needed to study the matrix effects. Our aim was to test the stability of this system by determining the degradation performance linked with the composition of both bacterial and fungal communities, as measured by DNA-fingerprinting techniques, in actual wastewater conditions.

## Materials and Methods

Wastewater was obtained from the wool dyeing process of Axminister Carpets, Devon, England, permission and certificate was granted from the owner Axminister Carpets. According to the dye master the wastewater contained several different azo and anthraquinone dyes together with support and process chemicals. Liquid Chromatography coupled with mass spectroscopy (LC/MS/MS) scans showed four peaks at RT 13.83, 15.28, 16.20 and 19.98 in the textile wastewater, which disappeared during decolorization (S1 Fig). The wastewater did not contain fat and waxes from the wash step for the wool. The wastewater had a pH of 4.7, a concentration of 960 mg L^-1^ volatile fatty acids (VFA), oxygen level of 68%, 41.1 mg L^-1^ nitrogen and the conductivity was 6750 μS/cm^3^ (approximately 4‰). The textile wastewater had a maximum absorbance at 594 nm and a COD of 3016 mg L^-1^. The water was transported by boat, approximately 4 days and divided into 3 L containers and frozen upon arrival. The transport may have affected the oxygen level and the concentrations of fatty acids, however there were no overpressure from gases in the container at arrival. The pH was modified to 6.5 with 5.5 mL 2 mol L^-1^ (11 mmol) sodium hydroxide per liter solution before the wastewater was fed to the bioreactors.

### Experimental design

**Fig 1:**
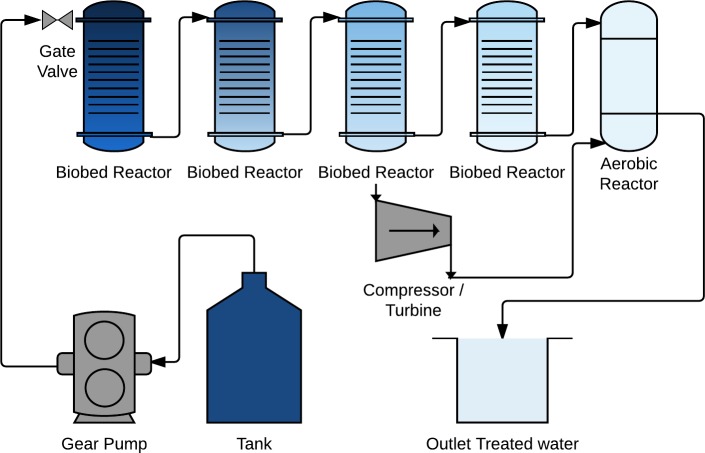
Experimental design. Biofilter set-up with anaerobic reactors R1, R2, R3, R4 and the aerobic R5 in series. The biobed in the reactors is supported by a net with a free water volume in the bottom.

The treatment facility was designed with several smaller reactors in a sequential series to monitor the degradation and microbial development in different parts of the biofilter and be able to change treatment volume and surface area for microbes. The biofilter had three anaerobic reactors (R1-R3) ø 40 mm x 300 mm, hydraulic volume of 300 mL, followed by one aerobic reactor (R5) ø 65 mm x 150 mm, hydraulic volume of 78 mL. After 29 days the treatment facility was extended with an additional anaerobic reactor (R4), to increase the hydraulic retention time in the anaerobic part and examine if it would affect degradation of metabolites from the dye, (Fig 1).

The experiment was run for 70 days with samples taken from the outlet of each reactor and analyzed weekly. The anaerobic reactors were filled to 260 mm (54.9 g) and the aerobic reactor to 40 mm with rice husks (5.0 g). Ang, Ngoh [25] reported that rice husks have an average surface area of 21.2 m^2^ g^-1^, a pore volume of 1.76x10^-2^ cc g^-1^ and a pore size of 33.2 Å. Rice husk is natural material that can differ in its structure, however Ang, Ngoh [25] values indicate a large surface available for microbes, if 1164m^2^ (54.9 g) is divided with the volume of the anaerobic part:

$$x=\frac{surface\;m^{2}}{\left({\left(\pi \;x\;radius\;m\right)}^{2}\;x\;hight\;m\;x\;4\right)}=\frac{1164m^{2}}{\left({\left(\pi \;x\;0,02m\right)}^{2}\;x\;0.30m\;x\;4\right)}=772\;000\;m^{2}/m^{3}$$ (1)

It indicates a treatment surface of the order of 772 000 m^2^/m^3^ in the anaerobic part of the filter. Including the aerobic part, the total surface available in the reactor was approximately 1270 m^2^ for the microbes to attach to in the biofilter. The system was fed with wastewater by a peristaltic pump (WatsonMarlow 400) with a flow of 22.3 mL h^-1^, resulting in a hydraulic retention time (HRT) of 13.4 h for each reactor, 54 h for reactors R1-R4 and 67 h including the aerobic reactor. Samples were taken from the outlet of each reactor.

### Chemical analyses

**Fig 2:**
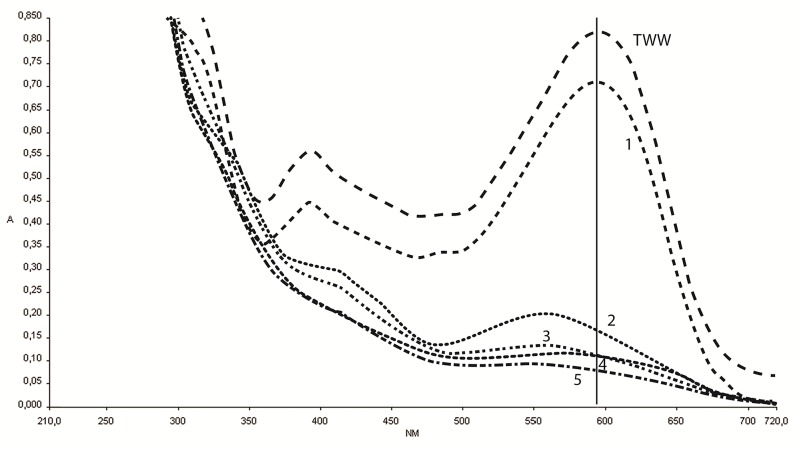
Microbial decolorization in the biofilter. Degradation of textile wastewater in reactors at steady state from 190 to 750nm; wastewater: TWW line, reactor 1: line 1, reactor 2: line 2, reactor 3: line 3, reactor 4: line 4, reactor 5 line 5. The vertical line marks the lambda max.

All samples were filtered through 0.45 μm filters (Sartorius AG, Goettingen, Germany) and scanned from 190 to750 nm with LAMBDA 35 UV/VIS spectrophotometer (Perkin-Elmer, Shelton, CT, USA) and analyzed using Perkin-Elmer UV WinLab software, version 2.85.04. The percent of degradation was calculated at the combined lambda max (594nm) for the wastewater (Fig 2):

$$\%\;microbial\;decolorization=100\;x\frac{absorbance\;wastewater-absorbance\;treated\;water}{absorbance\;wastewater}$$ (2)

The COD (LCK014, Hach Lange) and nitrogen (NH_4_-N, LCK303, Hach Lange) analyses were performed with Hach-Lange spectrophotometer DR2800 with heater block LT200. The samples were stored in a– 80°C freezer before analysis on LC/MS/MS and processing of DNA.

Liquid Chromatography coupled with mass spectroscopy (LC/UV/MS/MS) analyses were performed at the SP Technical Research Institute of Sweden with a MS-instrument: Quattro micro, Waters, equipped with electrospray-ion source, positive ion mode, capillary = 4 kV, cone voltage = 60 V, negative ion mode, capillary = 3.5 kV cone voltage = 60 V. The LC instrument had a: 2695 Separations module, Waters. column: Zorbax C18, 5 μm, 2.1 x 150 mm, and a Agilent UV detector Jasco UV-975 at 256 nm. The buffers used were: A = 50 mM formic acid with 3.03 mM Ammonium formate, B = Methanol, for programmed flow see [2]. To further examine if the treated water contained any aromatic amines, an acetylation with trifluoroacetic anhydride (TFAA) was performed: 10 μL samples were dissolved in 500 μl deionized water and 2 mL of solution chloroform/methanol (2:1, v/v) were added. Samples were mixed on vortex for 2 min and centrifuged at 7800 rpm, 5 min. Additionally, samples were evaporated by nitrogen flushing overnight and dissolved in 50 μL DuC/50 μL TFAA, incubated in 40°C for 2 h and evaporated overnight by nitrogen. Finally, each sample was dissolved in 500 μL acetonitrile.

### DNA collection and extraction

Collection of DNA and extraction were performed as done previously [2] with some modifications. 30 mL samples from the biofilters were filtered through a 47 mm, 0.2 μm pore-size polycarbonate filter (Poretics, Osmonics Inc. Minnetonka, MN, USA) at <200 mm Hg. The filters were immediately transferred to Cryovials (Nalgene, Thermo Fisher Scientific, Rochester, NY, USA), containing TE buffer (10 mM Tris, 1 mM EDTA, pH = 8.0), and frozen at -80°C until further processing. To break fungal cells we added a step with glass beads vortexing (1.5 + 1.5 min) [26]. A combined treatment with enzymes (lysozyme, proteinase K) and phenol/chloroform was used to extract the DNA as described in Riemann, Steward [27] but also using Phase Lock Gel Tubes^TM^ (5 Prime). Extracted DNA was re-suspended in TE buffer. In general, DNA concentrations ranged from 2–40 ng μL^-l^.

### Processing of bacterial and fungal amplicons

Bacterial 16S rRNA gene fragments were amplified using the primers GC341F with a 40 bp GC-clamp [28] and the universal primer 907R [29]. The PCR reaction was prepared with the PCR kit Illustra Ready To Go Beads (GE healthcare, Fairfield, USA) with 5–10 ng of DNA template. Initial denaturation was at 95°C for 2 min, followed by a thermal cycling program as follows: denaturation for 30 s at 94°C, annealing for 30 s at an initial 63°C, and decreasing 1°C every two cycles to a final of 53°C, extension for 90 s at 72°C. Ten cycles were run at 53°C for a total of 30 cycles followed by final 7 min incubation at 72°C. The PCR products were kept at 4°C. Fungal ITS region was amplified with PCR using the fungal primer ITS1f with GC clamp (primer ITS1f with a 40 base GC-clamp at the 5’ end: 5'CGC CCG CCG CGC GCGGCG GGC GGG GCG GGG GCA CGG GGG G CTT GGT CAT TTA GAG GAA GTA A 3’ [30] and ITS4 TCC TCC GCT TAT TGA [31]. The PCR reaction was prepared as above using Illustra Ready To Go Beads (GE healthcare, Fairfield, USA). PCR program for amplification of fungal DNA was as follows: initial denaturation at 95°C for 5 min; followed by 35 cycles of 94°C for 1 min, 55°C for 1 min, and 72°C for 1 min; and a final elongation at 72°C for 5 min. PCR products with the correct position and of good quality were purified with the E.Z.N.A. Cycle-pure Kit (Omega Bio-tek, USA). Denaturing gradient gel electrophoresis (DGGE) was performed as in [2]. The purified PCR products from re-amplification of DGGE bands were subsequently sent for sequencing at Macrogen (Netherlands). Sequences were controlled, corrected and edited using 4Peaks 1.7.2 [32]. Sequence identity was determined through a comparison with existing prokaryotic sequences for bacteria and eukaryotic sequences for fungi in GenBank (US National Center for Biotechnology Information) through BLAST (nucleotide—BLASTn). Phylogenetic tree was constructed using MEGA 5.2.1 [33]. Sequences have been deposited to GenBank under the accession numbers KF263975-KF263991 and KM347883-KM347888.

## Results and Discussion

### Dye decolorization

After 4 weeks of constant feeding with textile wastewater, the continuous sequential biofilter reactor system with five reactors (1–5) reached steady state. Subsequently, the organic compounds were degraded, and 90% of the color was removed in the anaerobic part (reactor 1–4). 58% of the COD was degraded by the whole system (HRT 67 h) at steady state. The setup was then run for an additional 6 weeks, 15 retention times (RT). In the beginning of the experiment, the rice husks beds (reactor 2–4) became slightly colored, a color that faded when steady state was reached. The biofilter performed a robust decolorization performance of the wastewater fed to the system during the whole experiment.

The color of the textile wastewater fed to the system was dark blue and most of the color was decolorized in the second reactor, while most of the COD was degraded in the aerobic reactor 5. Samples taken after 10 weeks (25 RT) showed that the wastewater passed the first reactor with only a slight color reduction whereas the color intensity dropped by 66% in reactor 2 (Fig 2). Concomitantly, the pH increased from 6.6 to 7.9 (Table 1) and 38% of the fatty acids were consumed.

**Table 1:** Characteristics of textile effluent and treated water.

	**Spectro**	**Decolorization**	**COD**	**Org Acids**	**pH**	**Oxygen**
	594 nm		mg L^-1^	mg L^-1^		
**Dye TWW**	0.82±	0%	3016±157	968±14	6.5±0,28	67.8%
**R1**	0.71±0,010	13.4%	2834±66	1128±179	6.63±,28	37.7%
**R2**	0.17±0,029	79.3%	2446±140	594±22	7.90±0,29	1.2%
**R3**	0.11±0,018	86.6%	2323±162	624±101	7.65±0,07	1.1%
**R4**	0.08±0,013	90.2%	2021±203	350±60	7.44±0,32	1.1%
**R5**	0.11±0,007	86.6%	1262±139	140±10	8.61±0,39	48.5%

Mean values for Textile wastewater (TWW) and samples from the outlets of each reactor: anaerobic reactor 1 (R1), anaerobic reactor 2 (R2), anaerobic reactor 3 (R3), anaerobic reactor 4 (R4), aerobic reactor 1 (R5), at steady state.

The color intensity continued to drop in the following anaerobic reactors, while the pH decreased slightly to 7.5. In the aerobic reactor 5, absorbance increased by around 4% and pH increased to 8.6. The COD concentration was only slightly reduced in the anaerobic reactors, where approximately 10% of the incoming COD was degraded, while an additional ~40% COD was reduced in the aerobic reactor. A test filtration of treated water through a small tube (15mL) of active coal absorbed the potential amine and the COD decreased to 173 mg L^-1^. A similar reduction was accomplished by another study that reported 44–52% COD reduction with different strains in actual textile wastewater [7]. Thus, our degradation of 58% of the COD in actual wastewater without any additional carbon source was comparable to or even improved.

When passing the first reactor the oxygen level decreased from 68% to 38%, further decreasing around 1% in reactor 2–4 and increased to 49% in the aerated reactor. The concentration of organic acids was unaffected in reactor 1, decreased by 38% in reactor 2 (from 949 to 594 mg L^-1^) and continued to decrease to 140 mg L^-1^ in the outlet of the aerobic reactor (Table 1).

LC/UV/MS/MS analysis to trace metabolites such as aromatic amines revealed that a majority of the background in the samples was composed of proteins and longer fatty acids. The LC/UV/MS/MS investigation did not show any signal related to aromatic amines (S1 Fig). By highlighting aromatic amines using a trifluoro-acetylation reaction, two possible aromatic amines were revealed in LC/UV/MS (S2 Fig), signals at the retention time (RT) 10.42 min and 25.40 min. The mass detection of the trifluoro-acetylated samples observed by UV at RT 10.42 min (S3 Fig) corresponds to two interesting masses observed in positive mode at m/z = 277.9 amu (prominent) and m/z = 392.0 amu. The ions detected are trifluoro-acetylated which increases the detected mass by 96 amu/trifluoroacetylation site as compared to the native compound. Furthermore, at RT 25.40 min, the trifluoro-acetylated signal detected by UV corresponds to ions at m/z = 297.2 amu and m/z = 389.2 amu (S3 Fig). Other UV-signals i. e RT = 1.12 and 26.63 min are also present in the background. Ions included in mass spectra in S3 Fig but not further discussed are due to background or not associated with relevant retention times. The LC/UV/MS/MS did not reveal any further information. The observed degradation products arose in reservoir two, increased during passage through reactor three and then decreased in reactor four and five. The signal at RT 25.40 min in UV-detection disappeared and the signal at RT 10.42 min decreased by 60% in the last two reactors.

LC/MS methods are sufficient to analyze aromatic amines from complex matrix and electrospray ionization source is described as one of the more sensitive methods [34]. Acetylation improves the resolution and increases sensitivity [35]. Taken together, our results suggest that the presence of aromatic amines in the treated samples were very low.

Concomitantly, the main part of the decolorization took place in reactor 2, which differed compared to our previous study on artificial wastewater [2], where the main decolorization took place in the first reactor. It is notable that degradation in artificially composed dye solutions compared to actual textile wastewater differs in performance in the same conditions. These findings suggest that studies on artificial solutions are important and useful but that it is crucial to test actual wastewater to develop sustainable and realistic biodegradation systems [36, 37]. Overall, the treatment system demonstrated a robust degradation performance, based on rice husks, a waste fraction readily available in developing countries.

### Microbial community composition

**Fig 3:**
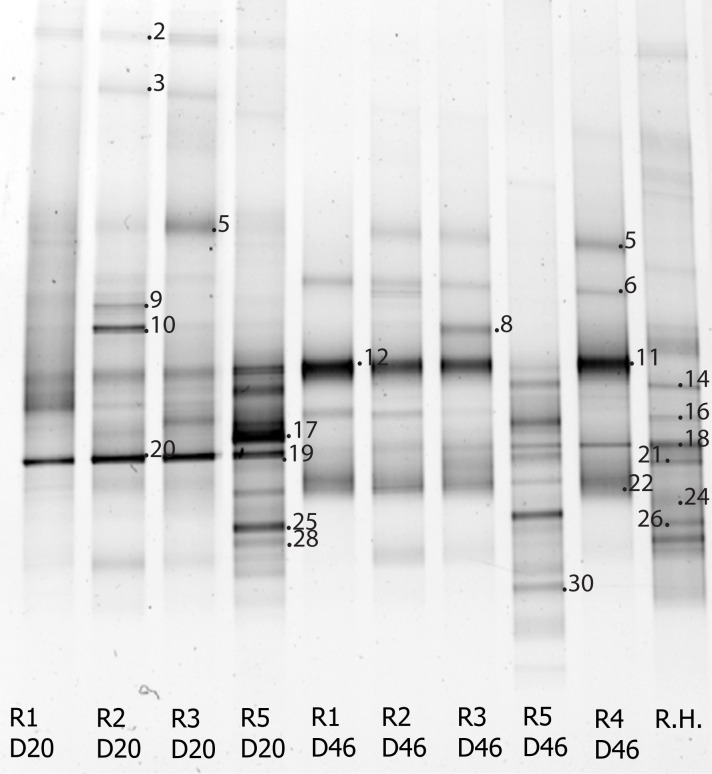
DGGE gel image of bacterial community composition in the experiments. Reactor 1–5 on day 20 (R1-R5 D20), reactor 1–5 on day 46 steady state (R1-R5 D46) and rice husks (R.H).

Microscopy revealed the presence of a variety of microbial cell types in the reactors. Notably, reactor one contained fungal hyphae, but very few bacteria in the samples. Additionally, samples from reactor 2–4 displayed bacterial aggregates. Analysis of DGGE fingerprints showed that there was a temporal shift in the microbial community composition concomitant with decolorization observed in the biofilters (Fig 3).

**Fig 4:**
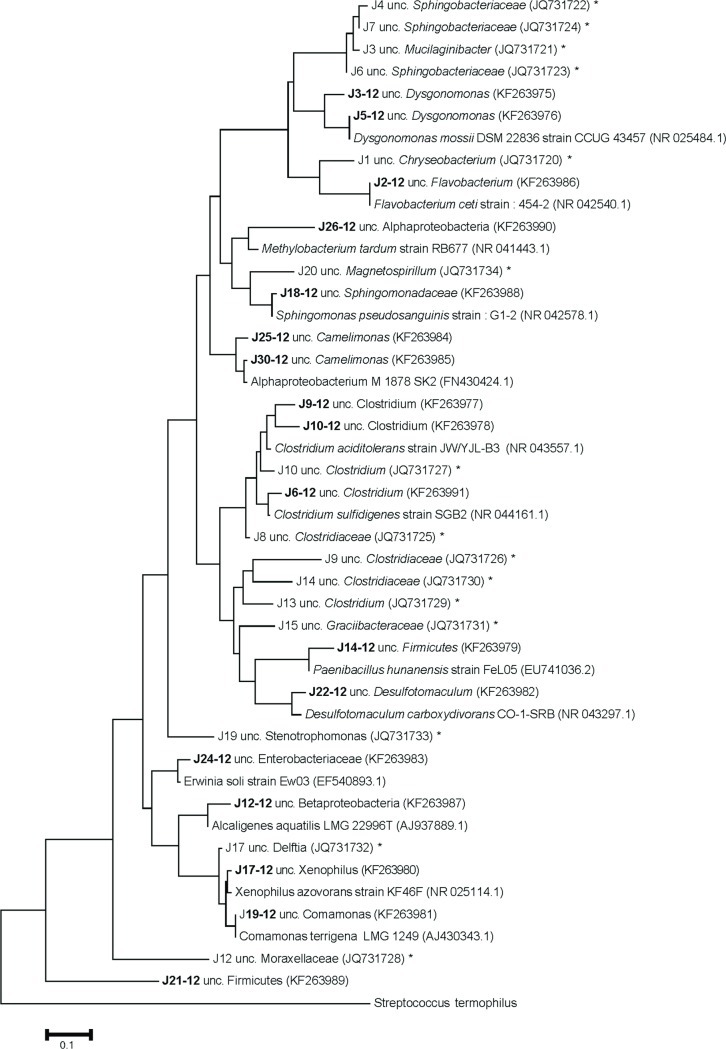
Phylogenetic tree of 16S rRNA gene sequences. Maximum-Likelihood based phylogenetic tree of 16S rRNA gene sequences obtained from DGGE bands and reference bacteria. Scale bar represent 0.1 nucleotide substitutions per site. Bold face text denote DGGE bands from this study and asterisks (*) denote phylotypes identified in (1). Parenthesis indicates GenBank accession number.

Typically between 5–8 abundant bacterial phylotypes (represented by strong bands in the DGGE gel) was observed in the anaerobic reactors. The banding pattern from the aerobic reactors showed a higher number of phylotypes, typically 10–14. Interestingly, reactor 1–4 displayed a similar bacterial community composition and the phylotypes had fairly similar relative abundances. In total, 19 phylotypes were sequenced, thus allowing for the annotation of particular microbial populations. At steady state, the first anaerobic reactor was dominated by *Clostridium* (Firmicutes) phylotype J6, *Alcaligenes* (Betaproteobacteria) phylotype J11, *Desulfotomaculum* (Firmicutes) phylotype J22 and *Pseudomonas* (Gammaproteobacteria) phylotype J16 (Figs 3 and 4). Interestingly, the *Dysgonomonas* phylotype J4 appeared in reactor 2, where a considerable decolorization was observed. Concomitantly, the *Acinetobacter* phylotype J8 was only present in reactor 3, and the *Sphingomonas* phylotype J18 was more pronounced in reactor 4.

The aerobic reactor had a different bacterial community composition compared to the anaerobic reactor, with 7 different phylotypes and only 4 phylotypes in common. For example, phylotypes J14 (*Paenibacillus*), J17 (*Xenophilus azovorans*), J21 (*Cohnella*), J25 (Alphaproteobacteria), J28 (unidentified) and J30 (Alphaproteobacteria) was unique in the aerobic reactors. At the same time, phylotypes J5 (*Dysgonomonas*), J6, J9, J10 (*Clostridium*), J11 (*Alcaligenes*) and J22 (*Desulfotomaculum*) had lower relative abundance in the aerobic compared to the anaerobic reactors. The indigenous community of rice husks (R.H.) carrying the inoculum, contained 8 relatively strong and 5–7 fainter bands. Phylotypes identified from rice husks were related with phylotypes present in both anaerobic and aerobic reactors (Fig 3).

Overall, the dominating phylotypes in the aerobic reactor were represented by bacterial taxa such as *Paenibacillus* (J14), Alphaproteobacteria similar to *Camelimonas* (J25, J30), *Xenophilus azovorans* (J17) and *Sphingomonas* (J18). Although several attempts were made to inoculate bacteria with wastewater solution (30 mL) from reactor 2 to reactor 1 to establish a bacterial community and boost degradation, no effect was observed. This was possibly due to fungal domination in reactor 1. Alternatively, the reactor had higher oxygen levels and lower pH. The microbial community pattern may also depend on the fact that most bacteria with azoreductase use electron shuttles to degrade the azo bond and oxygen inhibit this process by being a more potent electron acceptor [38]. Moreover, since the concentration of VFA decreased substantially in the second reactor it is likely that the microorganisms in this reactor utilized VFA as a carbon source.

The selective pressure in the biofilter promoted a bacterial community that displayed a variety of phylotypes with characteristics relevant for biodegradation of recalcitrant dyes. For example, *Clostridium sp*. is known to produce azoreductase and to degrade nitroaromatic compounds [39]. Also, *Acinetobacter* carry genes for azoreductases [40] and have proven to be efficient in degrading textile effluents [41]. Furthermore, *Pseudomonas* species carry genes for at least three different azoenzymes AzoR1, AzoR2 and AzoR3 [42]. *Sphingomonas*, here found in both anaerobic and aerobic biofilters, has genes for several azoreductases and has in earlier studies performed both successful dye degradation and mineralization of metabolites [43]. Even, *Xenophilus* has azoreductase capable of degrading several dyes with sulfonated naphtol ring structure, which is commonly considered recalcitrant. Notably, in this experiment *Xenophilus* had its strongest presence in the aerobic reactor implicating its preference for aerobic conditions. Another dominant bacterial phylotype in the biofilter (particularly in reactor R1-R4) was *Desulfotomaculum ruminis*, which has been whole-genome sequenced, yet no genes for azoreductase was found [44]. However, a close relative, *Desulfotomaculum orientis*, produce an FMN-dependent NADH azoreductase [40].

The order *Burkholderiales* was represented in the biofilter by two phylotypes: *Alcaligenes* (J11/J12) and *Comamonas* (J19/J20). It can be noted that several members of the *Comamonadaceae* family have been observed in wastewater treatment facilities [45], and are known to degrade recalcitrant compounds such as 4-nitrobenzoate [46] and sulfonated aromatics [47]. The *Alcaligenes* phylotype was predominant in the anaerobic parts of the investigated biofilter. *Alcaligenes* relatives have previously been found among microbial consortia degrading azo dyes [48]. Furthermore, the family *Alcaligenaceae* includes the species *Pigmentiphaga*, proven effective in decolorization of azo dyes [49], and has genes for at least two azoreductases [50]. Since most of the decolorization took place in reactor two, it is notable that bacteria related to *Alcaligenaceae* (J11), *Pseudomonas* (J16) and *Desulfotomaculum* (J22) had strong presence in this reactor. Several of the phylotypes found in the biofilters of this study have previously been pointed out as competent degraders of azo dyes. Thus, Verhagen [11] found several strains of Pseudomonas in biofilm structures and Saratale, Saratale [5] noted the presence of *Alcaligenes*, *Pseudomonas*, *Sphingomonas* and *Commomonas* is common in dye degradation studies. Collectively, the rice husks in the biofilter supported a varied set of bacteria likely to carry enzymes and capabilities that could potentially enhance biodegradation [5, 51] in actual textile wastewater.

### Fungal community composition

**Fig 5:**
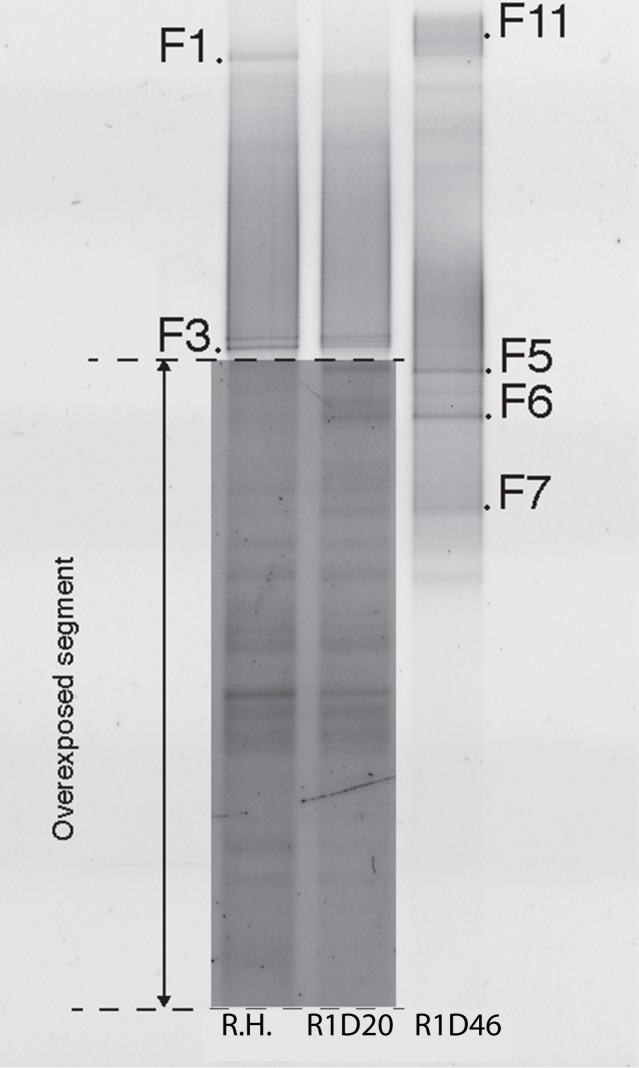
DGGE gel image of fungal community composition in the experiments. Reactor 1 (R1- D20), reactor 1 26 days later at steady state (R1-D46) and rice husks (R.H).

Microscopy analyses revealed fungal hyphae in reactor 1 and 5, and in accordance, fungi contributed to the microbial population in the rice husks and reactor 1. Eight different phylotypes were distinguished, and 6 of these phylotypes were sequenced (Fig 5, Table 2).

**Table 2:** Identification of sequenced Fungi ITS regions.

DGGE Band	GenBank accession numbers	Closest relative in Genbank	Phylum
F11	KM347888	Uncultured fungus clone 035A13854 (JX368294.1) (100%)	Asidomycota
		Uncultured fungus clone 035A11038 (JX365520.1) (100%)	
F6	KM347886	*Bullera* sp. TMS-2011 voucher SC11d50p13-2 (HQ631046.1) (99%)	Basidomycota
		Uncultured fungus clone 109A73680 (JX385442.1) (99%)	
F1	KM347883	*Fusarium andiyazi* strain CBS 134430 (KC954400.1) (96%)	Asidomycota
		*Gibberella intermedia* strain AR2_15 (JQ946368.1) (96%)	
F3	KM347884	*Gibberella intermedia* strain AR2_15 (JQ946368.1) (100%)	Asidomycota
		*Fusarium proliferatum* BLE1 (FN868470.1) (99%)	
F7	KM347887	Uncultured fungus clone 109A73680 (JX385442.1) (99%)	Basidomycota
		Uncultured fungus clone 109A73292 (JX385060.1) (99%)	
F5	KM347885	Hannaella sinensis strain CBS 7225 (AF444405.1) (100%)	Basidomycota
		*Bullera sinensis* strain JCM 5280 (AF325172.1) (100%)	

Incidentally, when we overexposed part of the DGGE gel containing samples from rice husks (R.H.) and reactor 1 at initial time point (R1-D20) there was a high number of bands visible indicating the presence of a wide variety of less abundant rare fungal phylotypes. Still, this diversity of bands was missing upon overexposure of the gel with samples from reactor 1 at steady state (R1-D46). Fungal phylotypes F1, F3, F11 were related to the genus *Fusarium*. Phylotype F1 was predominant in rice husks and present in reactor 1 at steady state but undetectable in R1-D20. Phylotype F11 was only detected in R1-D46, whereas phylotype F3 was present in both the rice husks and R1-D20. The phylotypes F5, F6 and F7, closely related to *Tremellaceae*, were all present in the rice husks and reactor one at steady state. Overall, there was a temporal development of fungal bands indicating that fewer phylotypes were established in the system at steady state compared to reactor 1 at the start of the experiment (Fig 5). The difference in fungal community composition between steady state and time 0 suggested an adaptation to the environment in the reactor where phylotypes related to *Fusarium* and *Trellaceae* had a selective advantage.

Interestingly, fungal phylotypes were detected in the aerobic reactor, but not in the anaerobic reactors, indicating that fungi required oxygen for growth. However, also reactor 5 had aerobic environment, but we could not successfully amplify fungal ITS gene fragments in this reactor. It is known that pH above 6.5 is disadvantageous for fungi and the biofilter had a pH between 6.5 and 8.6. Interestingly, the fungi identified in the biofilters belonged to the Ascomycota (phylotype F1, F3 and F11) and Basidomycota (phylotype F5, F6 and F7) phyla. Ascomycota contain fungal strains e.g. *Verticillium*, *Colletotrichum*, *Fusarium*, *Paecilomyces variotii* with potent dye degrading enzymes such as manganese-dependent peroxidases, ligninases and laccases [14]. Recently, the laccases have attracted substantial interest for biotechnological solutions [52]. In our study we detected three relatives of the *Fusarium* genus. This group of fungi has a documented ability to degrade polycyclic aromatic hydrocarbons (PAHs) [53]. For example *Fusarium oxysporum* utilize the β-ketoadipate pathway to degrade aromatic compounds [54]. Also, we detected three members of *Tremellaceae* known to carry enzymes, e.g. laccases, particularly suitable for degradation of aromatic compounds [54]. Furthermore, Basidomycota include several white-rot fungi, such as *Bjerkandera*, *Trametes versicolor*, *Pleurotus ostreatus*, that have displayed a capability to degrade different dyes [14]. Some species also produce several types of lignolytic enzymes such as laccase, lignin peroxidase and manganese peroxidase used in various applications in industry today to degrade pulp, dyes and other xenobiotics [52, 55]. It can be noted that the white rot fungi group carries several ligninolytic enzymes lignin peroxidase (LiP), manganese peroxidase (MnP), versatile peroxidase and Laccase. We note that these enzymes degrade phenolic and aromatic substrates through radical reactions with H_2_O_2_ under aerobic conditions. Collectively our results showed that fungi are present in the biofilters, and at least the rice husks and reactor 1 had representatives from two phyla that carry the potential to degrade aromatic compounds and textile dyes.

## Conclusion

Our findings emphasize that the indigenous microflora that developed in the bioreactors degraded both dyes and metabolites in actual textile wastewater with high efficiency. Previous studies using artificial dye solutions have indicated a promising potential for treatment of azo dyes under controlled conditions [2, 6, 56, 57]. The present study substantially expands on these findings, by constructing and investigating a biodegradation system using actual textile wastewater, which also included process chemicals. We show here that robust biodegradation by microbes from rice husks is achievable with such wastewater, and also discovered that similar bacterial species were found in the biodegradation systems with both artificial (see asterisks in Fig 4) and actual textile wastewater. These findings significantly extends previous results and highlight that the indigenous microbial flora of rice husks is robust and competitive, including fungi and bacteria with capabilities to degrade commercial textile dyes and other persistent compounds such as aromatic amines. Thus, a reliable degradation performance demonstrated here, constitute a commendable alternative to construct simple, affordable and applicable biotreatment of actual textile wastewater.

## Supporting Information

S1 Fig LC/UV of background, dye and treated samples. The dye from AxB displays three peaks, which degrades in R1- R2 mainly.
(TIF)

S2 Fig LC/UV of acetylated samples R1-R5. Trifluoro-acetylation reaction reveals two interesting peaks (possibly aromatic amines), with retention time 10.42 min and 25.40 min, arising when dye peaks were diminished (R1-R3). These formed peaks are then decreased in R4-R5.
(TIF)

S3 Fig MS spectra from peaks 10.42 and 25.40. The mass spectra from the peak with retention time (RT) 10.42 min corresponds to two interesting masses observed in positive mode at m/z = 277.9 amu (prominent) and m/z = 392.0 amu. Furthermore, at RT 25.40 min, the trifluoro-acetylated signal detected by UV corresponds to ions at m/z = 297.2 amu and m/z = 389.2 amu. The ions detected are trifluoro-acetylated which increases the detected mass by 96 amu/trifluoroacetylation site as compared to the native compound.
(TIF)
