# Supplementary figures and images for: Microbial Biotreatment of Actual Textile Wastewater in a Continuous Sequential Rice Husk Biofilter and the Microbial Community Involved
Source: PLoS One. 2017 Jan 23;12(1):e0170562. doi: 10.1371/journal.pone.0170562 (PMC5256951; doi:10.1371/journal.pone.0170562)

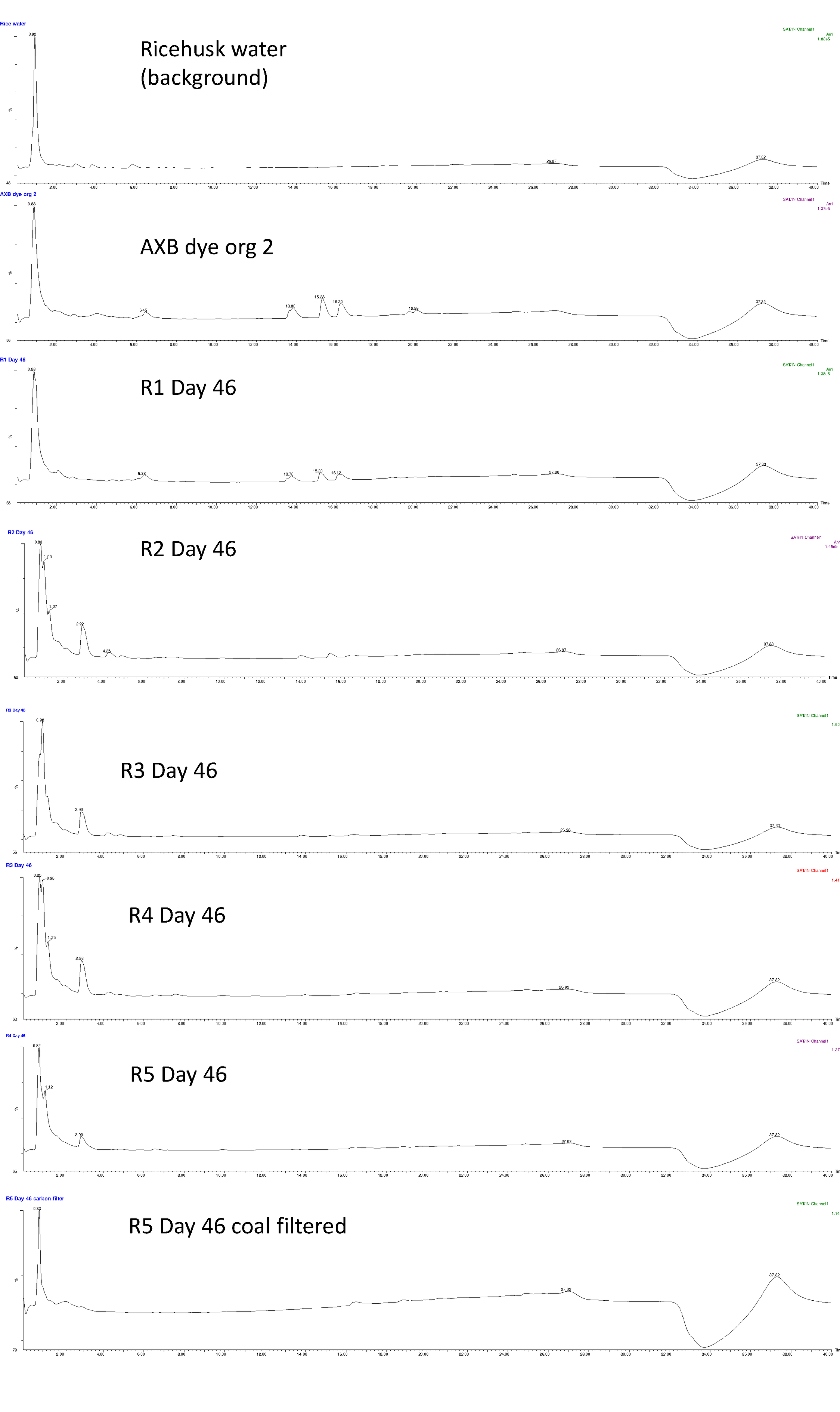

Supplement: S1 Fig — The dye from AxB displays three peaks, which degrades in R1- R2 mainly. (TIF) [file pone.0170562.s001.tif]

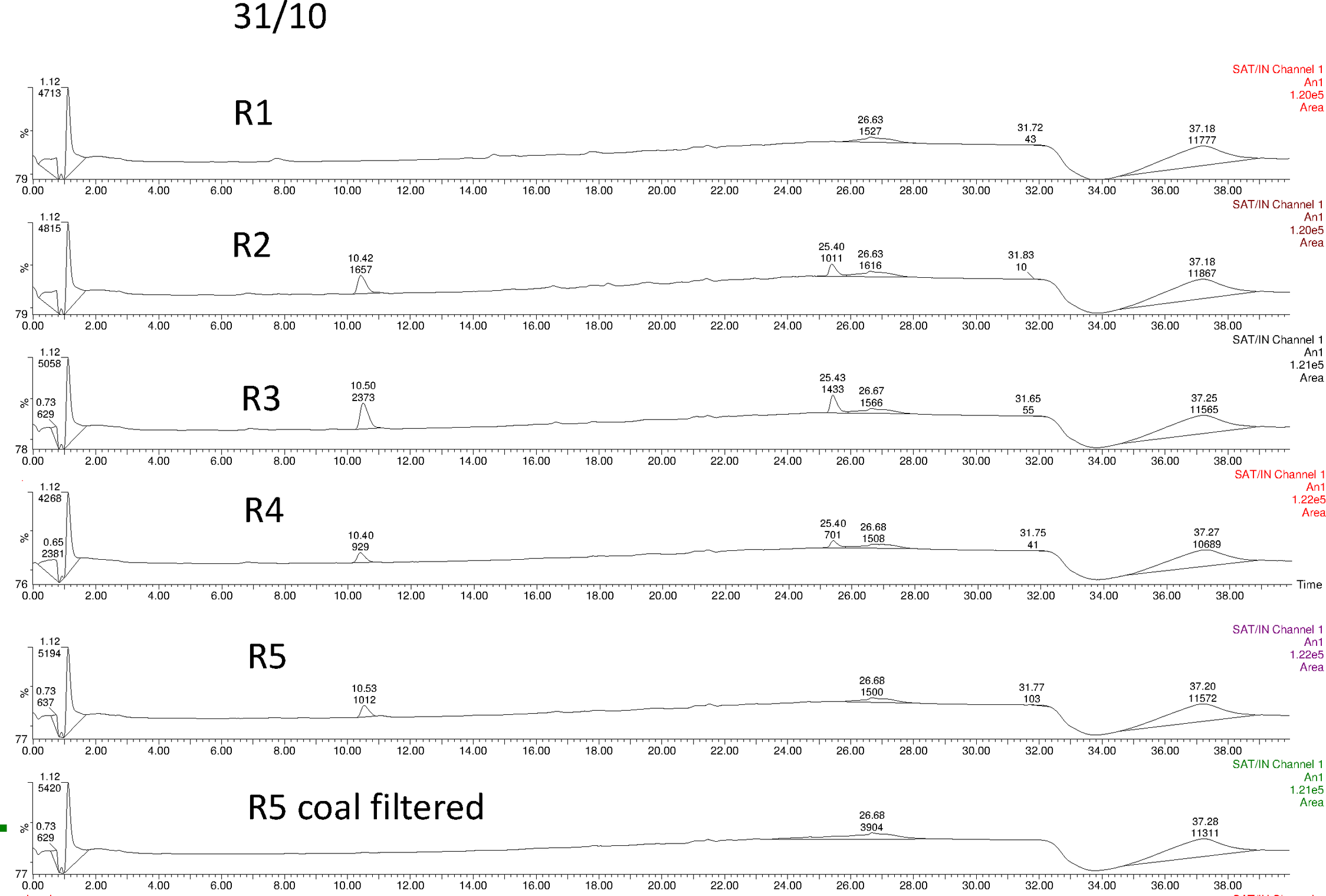

Supplement: S2 Fig — Trifluoro-acetylation reaction reveals two interesting peaks (possibly aromatic amines), with retention time 10.42 min and 25.40 min, arising when dye peaks were diminished (R1-R3). These formed peaks are then decreased in R4-R5. (TIF) [file pone.0170562.s002.tif]

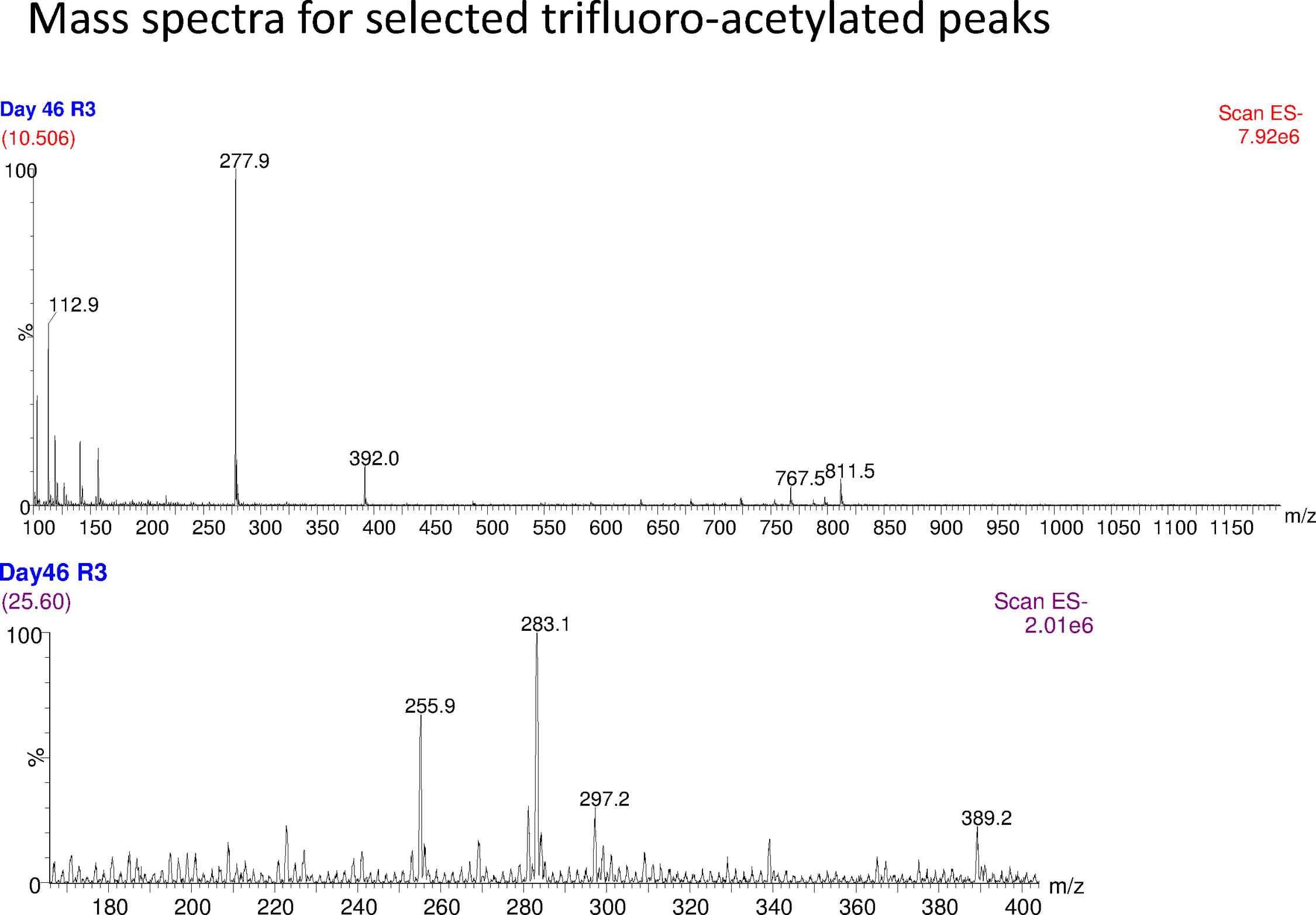

Supplement: S3 Fig — The mass spectra from the peak with retention time (RT) 10.42 min corresponds to two interesting masses observed in positive mode at m/z = 277.9 amu (prominent) and m/z = 392.0 amu. Furthermore, at RT 25.40 min, the trifluoro-acetylated signal detected by UV corresponds to ions at m/z = 297.2 amu and m/z = 389.2 amu. The ions detected are trifluoro-acetylated which increases the detected mass by 96 amu/trifluoroacetylation site as compared to the native compound. (TIF) [file pone.0170562.s003.tif]
